# CytoTree: an R/Bioconductor package for analysis and visualization of flow and mass cytometry data

**DOI:** 10.1186/s12859-021-04054-2

**Published:** 2021-03-22

**Authors:** Yuting Dai, Aining Xu, Jianfeng Li, Liang Wu, Shanhe Yu, Jun Chen, Weili Zhao, Xiao-Jian Sun, Jinyan Huang

**Affiliations:** 1 Shanghai Institute of Hematology, State Key Laboratory of Medical Genomics, National Research Center for Translational Medicine at Shanghai, Ruijin Hospital Affiliated to Shanghai Jiao Tong University School of Medicine and School of Life Sciences and Biotechnology, Shanghai Jiao Tong University, 197 Ruijin Er Road, Shanghai, 200025 China; 2 Division of Biomedical Statistics and Informatics, Department of Health Sciences Research and Center for Individualized Medicine, Mayo Clinic, 200 1st St SW, Rochester, MN 55905 USA

**Keywords:** Flow cytometry, Mass cytometry, Single-cell, Tree, Pseudotime

## Abstract

**Background:**

The rapidly increasing dimensionality and throughput of flow and mass cytometry data necessitate new bioinformatics tools for analysis and interpretation, and the recently emerging single-cell-based algorithms provide a powerful strategy to meet this challenge.

**Results:**

Here, we present CytoTree, an R/Bioconductor package designed to analyze and interpret multidimensional flow and mass cytometry data. CytoTree provides multiple computational functionalities that integrate most of the commonly used techniques in unsupervised clustering and dimensionality reduction and, more importantly, support the construction of a tree-shaped trajectory based on the minimum spanning tree algorithm. A graph-based algorithm is also implemented to estimate the pseudotime and infer intermediate-state cells. We apply CytoTree to several examples of mass cytometry and time-course flow cytometry data on heterogeneity-based cytology and differentiation/reprogramming experiments to illustrate the practical utility achieved in a fast and convenient manner.

**Conclusions:**

CytoTree represents a versatile tool for analyzing multidimensional flow and mass cytometry data and to producing heuristic results for trajectory construction and pseudotime estimation in an integrated workflow.

**Supplementary Information:**

The online version contains supplementary material available at 10.1186/s12859-021-04054-2.

## Background

Multidimensional flow and mass cytometric assays are widely used to identify cell subpopulations, determine tissue microenvironment compositions, perform clinical immunophenotyping and reconstruct differential lineages [1]. Modern fluorescence-based flow cytometers can detect up to 20 features, and their capacity can be increased to detect as many as 50 features with mass cytometers [2]. Traditional manual gating strategies can visualize flow cytometry data in only two or three dimensions [3], and effective visualizations and related workflows are still greatly needed [4]. With the rapid development of single-cell RNA sequencing (scRNA-seq), many computational workflows, such as Seurat [5], Monocle [6] and scmap [7], have been developed for scRNA-seq data. Unlike scRNA-seq data, however, flow and mass cytometry can focus on a subset of cellular markers or protein expression levels, producing data without many missing values [5]. The design of an scRNA-seq data analysis workflow could provide capabilities such as trajectory inference (studying the dynamic cellular processes [8]) and pseudotime estimation (reordering cells by their biological state to recapitulate the dynamics of biological processes [9]). The methods for trajectory inference and pseudotime estimation usually involve clustering, dimensionality reduction, and topological analysis based on a cell-to-cell network [8].

The data format flow cytometry standard (FCS) for both flow and mass cytometry data has been well established. For R users, tools such as flowCore [10] and OpenCyto [11], have been developed for reading and manipulating FCS data inputs and outputs, and provide basic functions for standardization, normalization and manual/automated gating control. Other existing tools, such as FlowSOM [12], SPADE [13] and PhenoGraph [14], can be used to classify the cells into different clusters. Dimensionality reduction algorithms for single-cell level data analysis were also repurposed for flow and mass cytometry data [15]. These include linear dimensionality reduction techniques such as principal component analysis (PCA) and nonlinear dimensionality reduction techniques, including t-distributed stochastic neighbor embedding (t-SNE, renamed viSNE) [16], diffusion maps [17] and uniform manifold approximation and projection (UMAP) [18]. Various clustering and dimensionality reduction algorithms make it difficult for users to test, compare, choose and evaluate for their data analysis. Furthermore, several emerging single-cell specific algorithms, such as developmental trajectory inference, lineage tracing and pseudotime estimation, have not been integrated into one analysis workflow with existing FCS tools. In accordance with well-established standards and practices [10, 12–14, 18–21], we present CytoTree, a trajectory inference, pseudotime estimation and visualization toolkit for flow and mass cytometry data. The CytoTree package is built in R and offers a completely up-to-date and feature-rich analysis workflow for flow and mass cytometry data, supporting a wide range of functionalities including classification, dimensionality reduction, trajectory construction, differentially expressed marker calculation, pseudotime estimation, intermediate state identification and visualization. To demonstrate these functionalities of CytoTree, we used two datasets, one for heterogeneity-based mass cytometry data and and the other for time-course flow cytometry data.

## Methods

### Generation of cytometry data

To generate time-course flow cytometry data, we involved the in vitro hematopoietic differentiation system from human embryonic stem cells (HUES9) to capture cells at different developmental stages. Specific antibodies used for staining hESCs-derived cells at different time were CD90 (BV421), CD49f (BV650), FLK1 (PE), CD34 (APC), CD31 (BV605), CD73 (BV711), CD43 (FITC), CD45(BV786-A), CD45RA (BV510), CD38 (PE-CY7). All antibodies were purchased from BD Biosciences. Brilliant Stain Buffer (BD Biosciences) was added to exclude staining artefacts that fluorescent dye interactions may cause. Compensation adjustments were made based on single stains. Flow cytometric analyses were performed on LSRFortessa (BD Biosciences). For mass cytometry of healthy human bone marrow, data was downloaded from FlowRepository database[22] (https://flowrepository.org/id/FR-FCM-ZY9R). This data was generated by Bendall et al. [23] and the preprocessing steps were implemented by Herring et al. [24]

### Preprocessing of the FCS data

**Fig. 1:**
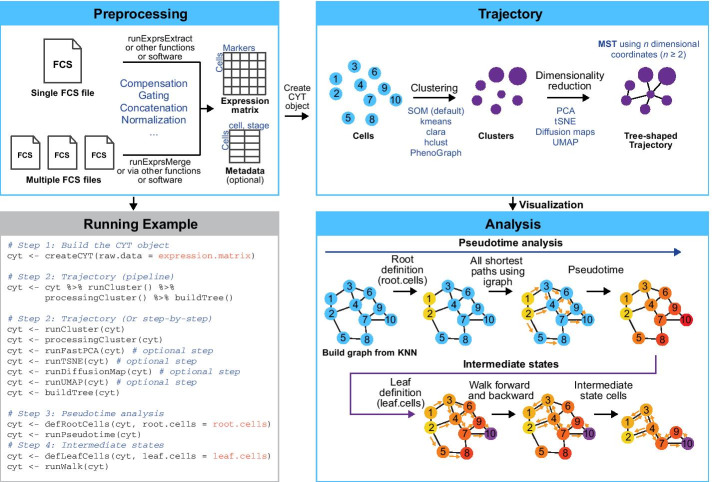
Overview of CytoTree package functionalities and algorithm. The preprocessing panel reveals the preparation steps before creating the CYT object. CytoTree provided functions to extract the expression matrix through a single FSC file or multiple FSC files. Both the clean expression matrix and meta-information are required to build the CYT object. The trajectory panel shows a the summary of the CytoTree workflow in constructing the tree-shaped trajectory. When the clustering was performed using all cells, all clusters of cells were linked by MST to illustrate the differentiation relationship based on the *n*-dimensional hull after dimensionality reduction. The analysis panel shows the model of pseudotime estimation and intermediate state identification. Each point represents one cell. A graph is built to connect all cells based on the KNN algorithm. Cells 1 and 2 (colored in yellow) are defined as the root cells. All the shortest paths from cells 1 and 2 to other cells are calculated to estimate the pseudotime. Cell 10 shows the maximum pseudotime and is then defined as the leaf cell (colored in purple). Forward and backward walks from the root cells and leaf cells are performed based on the shortest path. Cells 4 and 7 had the highest frequencies of occurrence during the walks and are considered to be the intermediate state cells. The running example panel shows the brief R code used to complete the entire workflow of CytoTree. Functions with “optional step” annotation are not the necessary steps in the CytoTree workflow

The preprocessing was facilitated by base packages obtained from Bioconductor, mainly flowCore [10] (Fig. 1, Additional file 1: Fig. S1, the preprocessing panel). CytoTree provides two functions that convert FCS files into an expression matrix, *CytoTree::runExprsExtract* and *CytoTree::runExprsMerge*. The two main steps that need to be implemented are compensation and filtration (also called gating). Compensation in the flow cytometry workflow has always been necessary. The manual adjustment of different fluorescence channels can be seen as the basic function of normalization and batch correction. Users could compensate the FCS files via the built-in functions in CytoTree. Regarding gating, CytoTree also provides functions to gate the matrix generated after compensation. We compared the scatter plot using FlowJo and CytoTree, and there was no difference (Additional file 1: Fig. S2), proving that CytoTree could be a reliable tool for performing compensation and gating on FCS data. If there was more than one sample in the study, such as a time-course study, a downsampling step was recommended to be preprocessed to ensure that the sample sizes at each time point were equal. In the case presented in this study, we used *CytoTree::runExprsMerge* to extract 2,000 cells at each time point and then merged them directly. A built-in function based on ComBat in the sva package [25] is integrated in the design of the CytoTree workflow for batch effect correction at different time points.

### Clustering in CytoTree

Five unsupervised methods were integrated into CytoTree (Fig. 1, Additional file 1: Fig. S1, the trajectory panel): self-organizing maps (SOM) [12], k-means clustering (kmeans) [26], clustering large applications (clara), PhenoGraph [14] and hierarchical clustering (hclust). Each method is independent and can be performed via *CytoTree::runCluster* by specifying different parameters. After clustering, cluster-dependent downsampling and dimensionality reduction were applied to each cluster. If the total cell sample size is over 100,000, it is better to perform downsampling to reduce the computational time. In the step of processing the clusters, four-dimensional reduction methods were applied to each cluster, including PCA, tSNE, diffusion maps and UMAP. The functions in the visualization part could be used to visualize and generate customizable, publication-quality plots. Visualization in CytoTree was mainly developed based on the R package ggplot2 (https://ggplot2.tidyverse.org/).

### Dimensionality reduction and trajectory reconstruction

Four methods (PCA, tSNE, diffusion maps, and UMAP) were integrated for dimensionality reduction enabling multidimensional data visualization in two or three dimensions. A trajectory could be constructed either from the expression profile or based on the dimensionality reduction coordinates; both were performed by the *CytoTree::buildTree* function. The trajectory construction was based on the minimum spanning tree (MST) algorithm [23] (Fig. 1, Additional file 1: Fig. S1, the trajectory panel). The use of the MST method in cytometry data was first proposed by Bendall et al. [23], and its accuracy, scalability, stability and usability were validated by Saelens et al. in scRNA-seq data [8]. To construct the trajectory, the coordinates of each cluster were first calculated. When using the expression matrix to construct the trajectory, the coordinates of the cluster were the expression value of each marker in the cluster.

$$Expression\,of\,marker\,i\,in\,cluster\,j = \frac{1}{n}\mathop \sum \limits_{k = 1}^{n} e_{i, j,k}$$

where $e_{i, j,k}$ is the expression of marker $i$ in cell $k$, cell $k$ is a cell in cluster $j$, and $n$ is the number of cells in cluster $j$.

When using the dimensionality reduction method to construct the trajectory, the coordinates of each cluster were the mean values of the coordinates of the cells in the cluster.

$$Coordinates\,of\,dimension\,i\,in\,cluster\,j = \frac{1}{n}\mathop \sum \limits_{k = 1}^{n} c_{i, j,k}$$

where $c_{i, j,k}$ is the coordinate of dimension $i$ in cell $k$, cell $k$ is a cell in cluster $j$, and $n$ is the number of cells in cluster $j$.

After all the coordinates of the clusters were determined, an undirected graph was built to connect all clusters based on the MST algorithm, which was invoked from the igraph package (https://igraph.org/). After the trajectory was built, branch analysis will be performed, and all clusters were divided into different branches according to the Louvain algorithm [5, 6]. The differentially expressed markers in each branch could then be calculated. The algorithm for calculating differentially expressed markers was based on the limma package [27], and this method was also integrated in the diffcyt package [28].

### Strategies for pseudotime estimation and intermediate state calculation

The algorithm used to estimate pseudotime was based on prior knowledge derivation. The estimation of pseudotime from the cell-to-cell shortest path was used in several algorithms and packages [29–31]. The steps in CytoTree to estimate pseudotime can be divided into four parts (Fig. 1, Additional file 1: Fig. S1, the analysis panel).

- Step 1: Define the root cells. A root cell is the initiation site of differentiation or the starting point of the biological process. The pseudotime in root cells was first set up to zero.
- Step 2: Construct a graph to connect all cells using the k-nearest neighbors (KNN) algorithm [32].
- Step 3: Calculate the distance from the root cells to all other cells by the shortest paths.
- Step 4: Calculate the pseudotime of each cell. The algorithm to transform distance to pseudotime was,
  $$\begin{aligned} Distance\,of\,cell\,i \left( {D_{i} } \right) & = \frac{1}{n}\mathop \sum \limits_{j = 1}^{n} dist_{i, j} \\ Pseudotime\,of\,cell\,i & = \frac{{D_{i} - Min\left( D \right) }}{Max\left( D \right) - Min\left( D \right) } \\ \end{aligned}$$
  where $dist_{i, j}$ is the shortest distance from cell $i$ to the cell $j$, cell $j$ is a root cell, and $n$ is the number of root cells. $D_{i}$ is the mean distance from cell $i$ to all root cells. $D$ is the set of $D_{i}.$

The root cells needed to be defined by users, and then the pseudotime was calculated. Users could identify the cell types based on marker expression and then set the ﻿initial cells as root cells. After pseudotime estimation, all cells were reordered by pseudotime and the KNN network could be modified based on pseudotime. When the pseudotime of cell $i$ was greater than that of cell $j$, the path from cell $i$ to cell $j$ could be accessed. To calculate the intermediate state cells, the leaf cells first needed to be defined first. The leaf cells were the terminal sites of differentiation. During the biological process, the differentiation was always multidirectional. The intermediate state cells were the cells that occurred were most likely to occur on the shortest path between the leaf cells and root cells based on the modified KNN network. Then, the leaf cells could be defined and used to estimate the intermediate state cells.

The source code and the detailed tutorial of CytoTree are all made available via our online tutorial https://ytdai.github.io/CytoTree/index.html.

## Results

### Overview of functionalities in CytoTree

The CytoTree package was developed as an analysis and visualization software for flow and mass cytometry data. Throughout the workflow, inputs/outputs, parameters and intermediate results are stored in an R S4 object (which we called “CYT”; Fig. 1). This design has two advantages, (i) it packages most computational approaches into an integrated analysis workflow, and (ii) it is convenient for users to adjust the parameters and obtain results. The main functionalities of CytoTree can be divided into four categories (Fig. 1, Additional file 1: Fig. S1): preprocessing, trajectory, analysis and visualization.

#### Preprocessing

This step involves data import, compensation, quality control, filtration, normalization and sample merging. The main aim is to prepare a clean expression matrix containing only clean cytometric signaling data for CYT object creation. CytoTree provides functions to deal with a single FCS file or multiple FSC files, such as in time-course experiments.

#### Trajectory

This step constructs a tree-shaped trajectory using the clean expression matrix prepared above. The first step in the trajectory part is clustering. All cells in the CYT object are grouped into clusters without supervision. CytoTree provides multiple methods for clustering, including SOM (by default), kmeans and many others. The second step is dimensionality reduction for both cells and clusters. The third step is constructing the tree that reveals subpopulation connections and cellular dynamic processes. In CytoTree, we use MST to construct the trajectory for all clusters.

#### Analysis

This step is designed for feature extraction and cell state refinement. Based on the tree built above, all branches are extracted to analyze the community structure of the trajectory topologies. The differentially expressed markers in each branch are identified, which can be further used to define the subbranches. For specific analysis of FCS data, such as tracing the cell of origin during differentiation and reprogramming, the pseudotime can be estimated. CytoTree provides algorithms and functions for pseudotime estimation and intermediate state cell identification (see Methods). It can help to reconstruct the processes of cell state changes based on dynamically expressed markers and provide an in-depth understanding of the differentiation and reprogramming process.

#### Visualization

This step offers various plotting functions to generate customizable and publication-quality plots. A two- or three-dimensional plot can be generated to illustrate the dimensionality reduction results, and a tree-based plot can be generated to visualize cell trajectories. Other special plots, such as heatmaps, marker density plots, specific scatterplots and violin plots, can also be generated in CytoTree for better interpretation and presentation of the data.

**Fig. 2:**
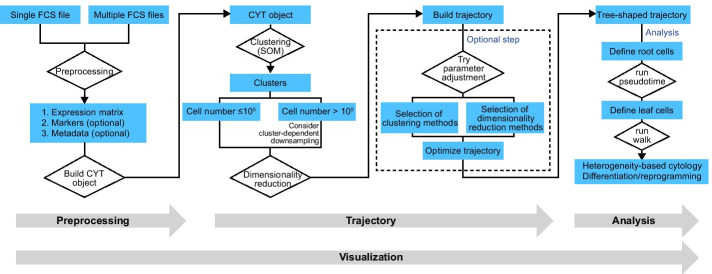
Summary of the analysis workflow of CytoTree. Summary of the CytoTree workflow for constructing the tree-shaped trajectory and performing pseudotime estimation and intermediate analysis. One single FCS file or multiple FCS files can be read and used to build the CYT object. The procedures of clustering, dimensionality reduction and the construction of tree-shaped trajectories can be run step by step. The optimization of the trajectory is an optional step consisting of the different combinations of clustering methods and dimensionality reduction methods. After that, we can define root cells and estimate the pseudotime, and then perform intermediate state analysis

CytoTree provides a clear and concise workflow for dealing with cytometry data in an effective and easy-to-comprehend manner. Figure 1 gives a graphical illustration of this workflow with the template codes included, while Fig. 2 summarizes the analysis workflow of CytoTree. After the CYT object was created, we integrated the most populated clustering method into CytoTree to make the analysis workflow more complete. Generally, the SOM clustering method is recommended, for better performance in precision, coherence, and stability than other unsupervised tools by Liu et al. [33] and Weber et al. [34] and also is recommended in the mass cytometry workflow [21]. At the dimensionality reduction step, it is recommended to perform cluster-dependent downsampling (if the cell number > 100,000) to reduce the computational runtime and speed up visual optimization. The trajectory is built from dimensionality reduction coordinates, allowing users to interpret the data by building the tree-shaped trajectory. If the trajectory constructed by the default parameters does not reflect the known biological process, CytoTree also provides the optimization step via parameter adjustment (Fig. 2). Users can construct the trajectory based on the expression matrix or by using the coordinates generated by the four dimensionality reduction, namely PCA, tSNE, diffusion maps and UMAP (Additional file 1: Fig. S3A, Fig. S3B). The default option is using the SOM clustering method and using the expression matrix. The performance of the tree-shaped trajectory calculated by different compensations of the clustering methods and dimensionality reduction methods was different (Additional file 1: Fig. S3B). The way of choosing the trajectory was based on two baselines: (i) whether the trajectory fits the real biological progress, and (ii) whether the tendency of the trajectory was maintained, which was determined by trying more combinations of the parameters of the cluster numbers, cluster methods, and dimensionality reduction methods. After the trajectory was built, the pseudotime estimation and intermediate state analysis could be performed for better interpretation of the biological process.

To demonstrate the practical utility of these functionalities in CytoTree, we introduce three use cases: (i) constructing a trajectory from single-cell mass cytometry data to recapitulate the human hematopoietic differentiation hierarchy, (ii) using the constructed trajectory to further analyze marker expression changes in different cell lineages during differentiation, and (iii) constructing the trajectory construction from time-course flow cytometry data to interpret the induced differentiation process of HUES9 cells.

### Use case 1. Identification of cellular subpopulations and construction of the trajectory to recapitulate the human hematopoietic differentiation hierarchy

**Fig. 3:**
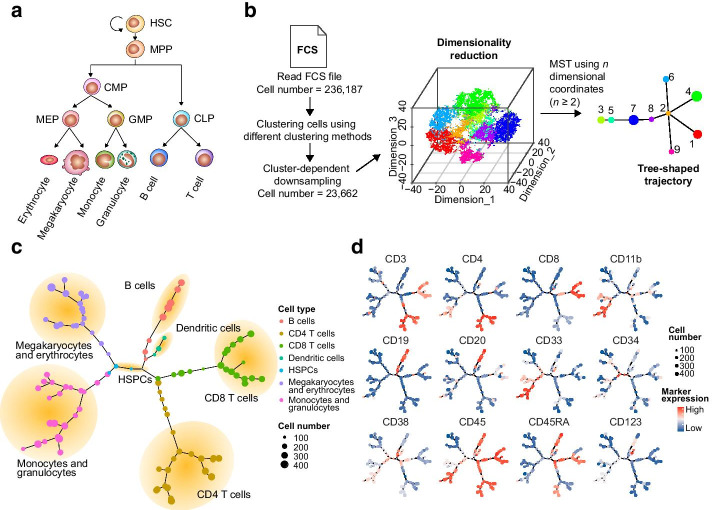
Analysis of mass cytometry data to identify the hematopoietic differentiation hierarchy. **a** Known hematopoietic hierarchy in the healthy human bone marrow. Hematopoietic stem cells (HSCs) are at the apex of the hematopoietic hierarchy and can produce progenitor cells, such as multipotent progenitors (MPPs), common myeloid progenitors (CMPs) and common lymphoid progenitors (CLPs). CLPs can give rise to T-lineage cells and B-lineage cells. CMPs can differentiate into either megakaryocyte-erythroid progenitors (MEPs) or granulocyte–macrophage progenitors (GMPs). MEPs can further differentiate into megakaryocytes and erythrocytes, whereas GMPs will become monocytes and granulocytes. **b** Workflow of constructing the tree-shaped trajectory using the 13-panel mass cytometry data. Raw FCS files were read using CytoTree. SOM clustering was performed using all cells. The cells were downsampled in a cluster-dependent fashion. The clusters of all cells were linked by MST to illustrate the differentiation relationship based on the *n*-dimensional hull after dimensionality reduction. **c** Tree plot with putative cell subpopulations annotated manually in yellow circles. The tree plot was constructed using 13 cell surface antigens present in healthy human bone marrow and revealed the hematopoietic differentiation lineages. The color of each cluster represents the branches identified. **d** Expression markers of CD4, CD8, CD11b, CD19, CD20, CD33, CD34, CD38, CD45, CD45RA, and CD123 expression markers are overlaid on the tree plot. The expression levels of the markers in each cluster were calculated by SOM clustering. These markers were used for the clustering to help define the different cell lineages

Through this case, in addition to demonstrating the power of cellular subpopulation identification and trajectory construction, we also intended to show two advantages of CytoTree: (1) the ability for data visualization and interpretation, and (2) the convenient and efficient workflow for cellular subpopulation analysis. To do this, we used a 13-marker panel mass cytometry dataset obtained from healthy human bone marrow cells [23]. As the hematopoietic differentiation hierarchy model represents a developmental relationship of the hematopoietic cells that reside in the bone marrow, the aim of this use case was to identify the cellular subpopulations and construct a tree-shaped trajectory, which could reveal the human hematopoietic differentiation hierarchy using the 13-marker panel mass cytometry data. According to the classical hematopoietic model, hematopoietic stem cells (HSCs) are at the apex of the hematopoietic hierarchy and can replicate themselves and produce precursors of various blood cell lineages [35, 36], such as megakaryocytes, erythrocytes, monocytes, granulocytes, B cells and T cells (Fig. 3a). In this use case, the raw FCS file was read and normalized using CytoTree. The raw FCS file contained 236,187 cells in total. After reading the FCS file and performing clustering by SOM, cluster-dependent downsampling was performed and only 10% of the cells were kept for further analysis and tSNE to build the tree-shaped trajectory and identify the cell types in different branches (Fig. 3b, Additional file 1: Fig. S4). According to the marker expression of each cluster, the cell type and differential lineage could be identified based on the trajectory (Fig. 3c). By using the tree plot of the trajectory, hematopoietic stem/progenitor cells (HSPCs, including HSCs and progenitor cells) could be identified by the expression of the marker combination CD45^+^CD34^+^CD3^−^CD19^−^ (Fig. 3c, d). Branch analysis revealed two different lineages of hematopoietic cells according to the trajectory: lymphoid lineage cells, including T cells and B cells and myeloid lineage cells, including monocytes and granulocytes, megakaryocytes and erythrocytes (Fig. 3c). In the lymphoid lineage branch, the subpopulations of the T-cell lineage, including CD4 T cells and CD8 T cells, showed the characteristic markers CD45^+^CD3^+^ (Fig. 3d). Regarding other lymphoid cells, the surface markers of B cells were CD45^+^CD3^−^CD19^+^CD20^+^. In addition to the B-cell branch, a small branch with high CD123 expression indicated the dendritic cells. For the myeloid lineage branch, monocytes and granulocytes showed the characteristic markers CD45^+^CD3^−^CD19^−^CD33^+^CD11b^+^, and megakaryocytes and erythrocytes were negative for these markers (CD45^−^CD45RA^−^) (Fig. 3d). In summary, we used CytoTree to identify hematopoietic cellular subpopulations and to construct a tree-shaped trajectory, recapitulating relationships that are consistent with the classical hematopoietic hierarchy model.

### Use case 2. Analysis of marker expression to illustrate the dynamic process of cell lineage differentiation

**Fig. 4:**
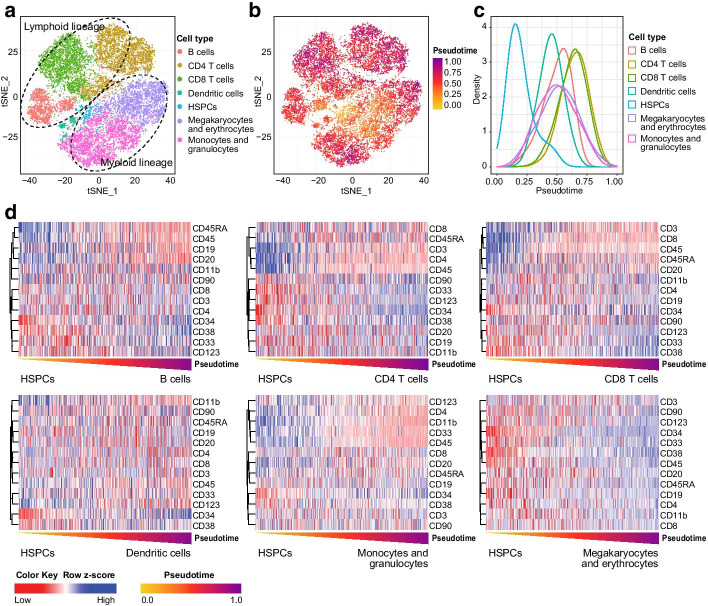
Pseudotime estimation and the identification of intermediate states in the hematopoiesis of different cell lineages. **a** tSNE visualization of the cells in each branch. The cells are colored according to the branches identified in use case 1. **b** tSNE visualization of the cells according to the hematopoietic differentiation process identified in use case 1. The cells are colored according to the pseudotimes calculated in CytoTree. **c** Density plot of the pseudotime for different cell lineages. **d** Heatmap plot of the intermediate state cells along with the pseudotime progression. Each column represents a cell. The expression values of the intermediate cells of each lineage are scaled by row and visualized using the z-score

Through this case, by using a workflow including the progress of pseudotime estimation and intermediate state identification, we illustrate the framework for heterogeneity-based cytology experiments using CytoTree and show two advantages: (1) pseudotime estimation is applicable, and (2) cells in the intermediate state reveal key markers during differentiation. HSCs are at the apex of the hematopoietic trajectory, and their differentiation is multidirectional [35]. For differentiation-based research, reconstructing lineage relationships and studying the dynamic process during differentiation are important. To infer the cells of origin of different blood cell lineages from a tree-shaped differentiation trajectory, the times of all cells were deconformed and rebuilt, which is also called pseudotime estimation. In this use case, we used the same dataset from use case 1 above to estimate the pseudotime for mass cytometry data and to trace the origins of cells according to the trajectory. Based on the tree-shaped trajectory, we first defined HSPCs as root cells according to the classical hematopoietic differentiation hierarchy model [35]; they were placed in the middle and surrounded by various blood cell lineages (Fig. 4a). Based on the results of the pseudotime estimation, the cells that were closer to the HSPCs had earlier pseudotime (Fig. 4b). According to the distribution of the pseudotime of all blood cell lineages, HSPCs had the earliest pseudotime, whereas dendritic cells, monocytes and granulocytes, megakaryocytes and erythrocytes followed closely behind (Fig. 4b). The lymphoid cells differentiated immediately after, and CD8 T cells were last (Fig. 4c). To illustrate the dynamic differentiation processes of different blood cell lineages, we set the terminal clusters of the different branches of the trajectory as the leaf cells and then used CytoTree to identify the intermediate state cells. The marker expression changes during pseudotime progression revealed the dynamic processes of the different cell lineages. For example, during the formation of B cells, the expression levels of CD19, CD20, CD45 and CD45RA were increased. During the development of CD8 T cells, the expression levels of CD8, CD45 and CD3 were increased during pseudotime progression, and for CD4 T cells, CD4, CD45 and CD3 were increased (Fig. 4d). For dendritic cells, CD123 was increased during differentiation (Fig. 4d). Monocytes and granulocytes, it showed increases in CD11b and CD45, whereas all markers of megakaryocytes and erythrocytes showed decreases (Fig. 4d). In summary, this use case illustrated how to calculate pseudotime and how to identify intermediate state cells using CytoTree. Overall, CD34 expression was gradually decreased and was mutually exclusive for markers such as CD4, CD8, CD19, CD20 and CD11b. Through pseudotime estimation, we could better understand the biological progression during hematopoiesis and the hypothetical marker changes occurring during the formation of different blood cell lineages. CytoTree demonstrated its advantages during the analysis of use cases 1 and 2. We used trajectory construction to reveal the hematopoietic differentiation hierarchy and then performed pseudotime estimation to reveal the differentiation stages of the blood cell lineages during hematopoiesis. Through the identification of the intermediate state cells, differences in marker expression could be used to illustrate biological changes in cells and to analyze the formation of different cell lineages.

### Use case 3. Differential trajectory construction using time-course flow cytometry data

**Fig. 5:**
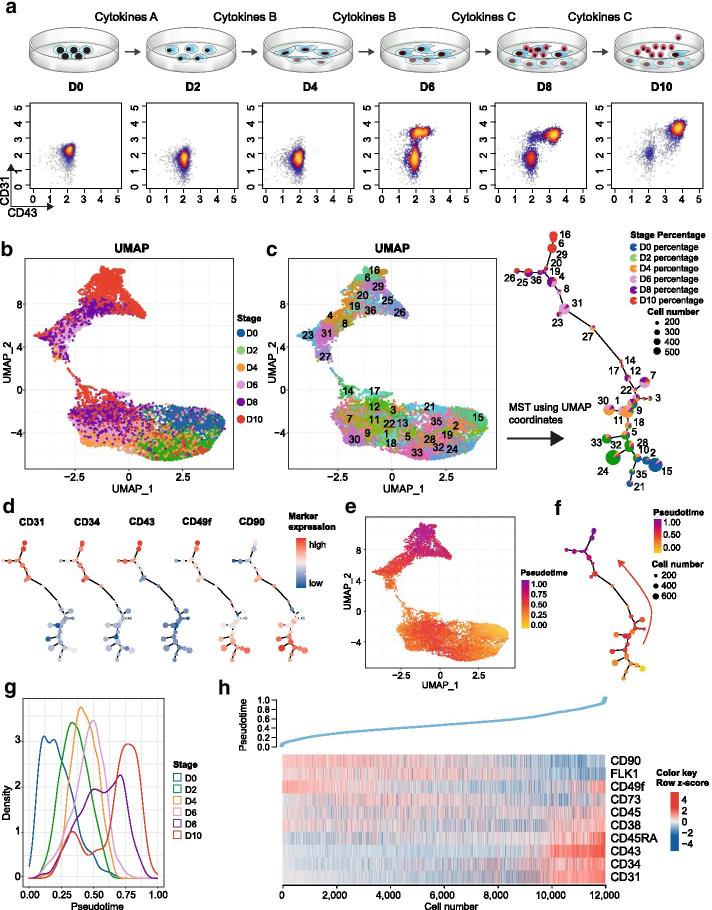
Analysis of time-course flow cytometry data reveals the induced differentiation process. **a** Experimental workflow and kinetics of CD43 and CD31 expression during the hematopoietic differentiation of HUES9 cells from D0 to D10. HUES9 cells were directionally differentiated into mesodermal cells (D4, cells with flattened shapes), hemogenic endothelium (D6, cells with squamous shapes) and HSPCs (cells in red) in succession. **b** UMAP visualization of the merged cells from the hematopoietic differentiation process. Cells are colored according to the time point from D0 to D10. **c** Construction of the hematopoietic differentiation trajectory based on UMAP coordinates using MST. **d** CD31, CD34, CD43, CD49f and CD90 expression markers are overlaid. **e** UMAP visualization of cells according to the hematopoietic differentiation process. Cells are colored according to the pseudotime. **f** Differentiation tree colored according to pseudotime. The cluster color is scaled to the mean value of the pseudotime for the cells within each cluster. **g** Density plot of the pseudotime for different differentiation days. **h** Heatmap of marker expression along with pseudotime progression. Each column represents a cell. The expression values are scaled by row and visualized using the z-score

In this case, by illustrating the usage of differential trajectory reconstruction of time-course FCS data, CytoTree shows two advantages: (1) the ability to reveal real induced differentiation progress with the tree-shaped trajectory, and (2) the ability to reveal biological changes and cells of origin via the analysis of differential experiments. This use case also provides a framework for time-course cytometric data analysis and could provide support for research on stem cell reprogramming. In this case, we used a flow cytometry dataset of ten-day hematopoietic differentiation from the hESC line HUES9 on the basis of some modifications of the previous work [37]. By adding different cytokines on different days, HUES9 cells (cells on Day 0, D0) were directionally differentiated. Mesodermal cells (D4), hemogenic endothelial cells and hematopoietic stem/progenitor cells (HSPCs, cells generated on D8) were generated from HUES9 cells in succession (Fig. 5a, Additional file 1: Fig. S5). Ten cell surface markers (CD90, CD49f, FLK1, CD34, CD31, CD73, CD43, CD45, CD45RA, and CD38) were used for flow cytometry analysis to monitor the generation of these cells. In particular, the initial expression of CD31 and CD43 at D6 and D8, respectively, reflected the emergence of endothelial cells and the endothelial-to-hematopoietic transition (EHT) (Fig. 5a, Additional file 1: Fig. S5). The aim of this use case was to reconstruct the cellular differentiation trajectory of HUES9 cells and identify the cell of origin of HSPCs using CytoTree. For this use case, the FCS file for each time point was filtered and compensated independently to exclude variance caused by unstable voltages from different experiments. Manual compensation correction was performed based on isotype control. After compensation and gating, 2,000 cells from each time point were collected and merged together to build the object. By using the default parameters, 36 clusters were identified using the SOM algorithm. After processing the clusters, the time-course cytometric data were visualized and interpreted in two or three dimensions using the four dimensionality reduction methods in sequence (Additional file 1: Fig. S6A). Based on the dimensionality reduction results, the two-dimensional UMAP visualization method obtained results that best fit the real-time biological processes (Fig. 5b, Additional file 1: Fig. S6A, Fig. S6B). In this use case, we used UMAP coordinates to build a tree-shaped trajectory through the MST (Fig. 5c). As both shown by both the tree-shaped trajectory and cluster-based dimensionality reduction results, the cells at each time point were gradually distributed along with the branch extension and were differentiated into two directions at the terminal state (Fig. 5c). Together with the marker expression for each cluster, the cells in clusters 18 and 12 showed the characteristic markers CD90^+^CD49f^+^CD34^−^CD43^−^, and the majority of the cells were on D0 (Fig. 5d), revealing that these clusters were HUES9 cells and were in the initial state of differentiation. Branch analysis was then performed to identify the cellular subpopulation composition, and six branches were clustered (Additional file 1: Fig. S7A). Branch A comprised HUES9 cells, branches B and C included FLK1^+^ mesodermal cells, and branch D comprised hemogenic endothelium expressing the markers CD34^+^CD31^+^CD43^−^. At the end of the trajectory, the clusters were divided into two different branches, branches E and F (Additional file 1: Fig. S7A, Fig. S7B); one branch was characterized by the markers CD31^+^CD34^+^CD43^+^CD49f^−^, and the other branch expressed the markers CD31^+^CD34^+^CD43^+^CD49f^+^. The two branches showed the characteristic markers of hematopoietic progenitor cells (HPCs, CD49f^−^) and hematopoietic stem cells (HSCs, CD49f^+^) (Fig. 5d and Additional file 1: Fig. S7B). This result was consistent with our previously induced hematopoietic differential model of HUES9 cells and suggested CD49f as a determining factor for the terminus of differentiation [38] (Fig. 5d). In summary, by constructing the induced differentiation trajectory of HUES9 cells using CytoTree, this use case demonstrates the utility of CytoTree for investigating cell-to-cell heterogeneity during induced differentiation.

After completing the cellular subpopulation identification and differentiation lineage reconstruction, a subpopulation of cells could be identified as root cells that were almost at the origin on D0 and had a marker composition similar to that of hESCs according to prior knowledge (Fig. 5d). We estimated the pseudotime by considering the cells in cluster 15 as root cells (Fig. 5c). After the pseudotime for each cell was calculated, the pseudotime distribution was found to be consistent with the UMAP visualization plot and remained consistent with the real-time change (Fig. 5e–g). By analyzing the correlation between the pseudotime and marker expression, we observed that CD45RA, CD43, CD34 and CD31 were increased and CD90 was decreased, whereas FLK1 increased at first and then decreased during pseudotime progression (Fig. 5h, Additional file 1: Fig. S8). In summary, this use case illustrates how to construct the trajectory and calculate the pseudotime using time-course FCS data. Through pseudotime estimation, we could better understand the biological changes occurring during the induced differentiation of cells and reveal which types of markers or cells might play the most important roles in this process.

### Comparison to other software tools and workflows used for flow and mass cytometry data analysis

We compared the functionalities between CytoTree and other commonly used software packages and workflows including OpenCyto [11], MetaCyto [39], FlowIO [40], FlowCal [41], AutoGate [42], cytofkit [14], diffcyt [28], CytoSOM (https://github.com/gautierstoll/CytoSOM) and CyTOF workflow [21] (Table 1). In preprocessing, all methods support the filtration, concatenation and normalization of FCS data. For the clustering, CytoTree has integrates the greatest number of clustering methods. To calculate the features of the clusters, both the CyTOF workflow [21] and CytoTree provided a clustering processing step to select the features of the clusters identified in the previous step. After constructing the tree-shaped trajectory, CytoTree provides unsupervised clustering on the clusters to define branches. For the dimensionality reduction procedure, four methods are integrated into CytoTree workflow, and these methods have been widely used in current single-cell analysis workflows. In these packages and workflows, the CyTOF workflow and CytoTree integrated the UMAP method. In addition, cytofkit [14], CyTOF workflow and CytoTree provided functions to perform cluster-based downsampling to avoid mistakenly dropping small clusters. Unlike other tools and workflows, CytoSOM and CytoTree provided functions to construct a tree-shaped trajectory based on MST. CytoTree also provided functions to optimize the tree, e.g. constructing the trajectory using different dimensionality reduction coordinates. Of special note is that for time-course data analysis, CytoTree provides pseudotime estimation to analyze the experimental data obtained during induced differentiation as well as functions to determine the intermediate state cells and to identify their expression characteristics. Thus, CytoTree provides a complete analysis workflow for flow and mass cytometry data based on a variety of methods used in other software tools and workflows, and it also integrates algorithms to estimate the pseudotime and to define the intermediate state cells according to time-course data. In summary, CytoTree is a competitive workflow that can comprehensively analyze flow and mass cytometry data, featuring user-defined parameters and styles in the construction of tree-shaped trajectories and pseudotime estimation.

**Table 1:** Comparison between other tools and workflows and CytoTree

Computational modules	Functions	OpenCyto	MetaCyto	FlowIO	FlowCal	AutoGate	cytofkit	diffcyt	CytoSOM	CyTOF workflow	CytoTree
Preprocessing	Denisty plot	√	√	√	√	√	√			√	√
	Gating	√	√	√	√	√		√	√	√	√
	Concatenation	√	√	√	√	√	√	√	√	√	√
	Normalization	√	√	√	√	√	√	√	√	√	√
Clustering	SOM		√				√	√	√	√	√
	kmeans		√							√	√
	clara										√
	PhenoGraph						√			√	√
	hclust										√
Processing clusters and branches	Downsampling						√			√	√
	DR-clusters					√		√		√	√
	Diff-exp					√		√	√	√	√
Dimensionality reduction	PCA						√			√	√
	tSNE						√			√	√
	Diff-maps										√
	UMAP									√	√
Trajectory	Tree								√		√
Analysis	Pseudotime										√
	Inter-state										√

*DR-clusters* dimensionality reduction for clusters, *diff-maps* diffusion maps, *diff-exp* differentially expressed markers, *inter-state* intermediate state analysis

### Computational scalability and analysis framework of CytoTree

In terms of computational scalability, CytoTree has been developed as a lightweight software package and can process workflows involving as many as 20,000 cells and 13 markers in less than 10 min on a MacBook platform with 8 Gb of RAM and a 2.7 GHz Dual-Core Intel Core i5. To improve the runtime and performance of CytoTree, we tested a series of simulated data with different cell numbers and marker numbers (Table 2). The simulation tests were performed on a LINUX platform with a 56-core Intel(R) Xeon(R) CPU E5-2695 v3 @ 2.30 GHz with 512-GB RAM. Only one thread was used when running the tests. CytoTree can handle up hundreds of thousands of cells in all computational parts. It took a great amount of time for dimensionality reduction, such as running tSNE, obtaining diffusion maps, and performing UMAP (Table 2), but the dimensionality reduction step is optional. For example, when the cell number was 100,000, it took more than an hour to complete all processes. Without running this optional step, it would take less than ten minutes.

**Table 2:** Running time of different functions in CytoTree with different cell numbers and marker numbers

Functions in CytoTree	C 1*10^3^ M 10	C 1*10^3^ M 50	C 1*10^4^ M 10	C 1*10^4^ M 50	C 5*10^4^ M: 10	C 5*10^4^ M 50	C 1*10^5^ M 10	C 1*10^5^ M 50	C 5*10^5^ M 10	C 5*10^5^ M 50
createCYT	1s	1s	1s	1s	1s	1s	1s	2s	2s	2s
runCluster	1s	1s	1s	1s	2s	2s	2s	4s	5s	16s
processingCluster	1s	1s	1s	1s	2s	2s	2s	2s	2s	2s
runFastPCA*	1s	1s	1s	1s	2s	2s	2s	3s	9s	13s
runTSNE*	4s	4s	7s	1m35s	5m54s	13m22s	13m52s	36m30s	2h11m19s	10h57m17s
runDiffusionMap*	1s	2s	21s	36s	8m8s	18m42s	36m54s	1h35m15s	16h16m33s	> 2 days
runUMAP*	5s	7s	1m32s	2m32s	9m17s	20m28s	21m35s	51m46s	2h17m28s	7h57m58s
buildTree	1s	1s	1s	1s	2s	2s	2s	2s	4s	8s
defRootCells	1s	1s	1s	1s	1s	1s	1s	1s	2s	2s
runPseudotime	1s	1s	9s	13s	3m17s	6m25s	5m46s	17m9s	12m41s	2h58m21s
defLeafCells	1s	1s	1s	1s	1s	1s	1s	1s	2s	2s
runWalk	1s	1s	7s	7s	2m1s	1m48s	2m1s	1m51s	2m18s	2m30s
Total time (all steps)	19s	22s	2m23s	5m10s	28m48s	1h56s	1h20m19s	3h22m46s	21h45s	> 3 days

*C* cell number, *M* marker number, *3h22m46s* 3 hours 22 minutes and 46 seconds. Functions with * were optional steps, which are not the necessary steps in the CytoTree workflow. Calculation of total time contains all steps, including the optional steps

## Discussion

CytoTree was capable of performing clustering, dimensionality reduction, trajectory inference and pseudotime estimation, which collectively forming an integrated workflow for flow and mass cytometry data analysis. During the analysis of time-course flow cytometry data in use case 3, batch effects in FCS data are usually caused by unstable voltages and different experimental environments or instruments. To minimize the impact of batch effects, we collected a cell sample at a specific time every two days to ensure that the interval times between each time point were equal and performed manual compensation correction based on the isotype control. However, the batch effect was unavoidable. Effective and appropriate methods to correct for the batch effect in flow and mass cytometry data are still needed. Therefore, it is recommended to perform batch effect correction in the preprocessing step prior to building the CYT object. Using a clean matrix instead of raw FCS files as input data can increase the expansibility of CytoTree.

In the dimensionality reduction procedure, the expression matrix was used for the tSNE and UMAP calculations, which is different from scRNA-seq analysis software such as Seurat [5] and Monocle [6]. This design is motivated by the fact that the number of markers of mass cytometry data is much less than that of scRNA-seq. There are several public trajectory inference strategies for single-cell data, such as PAGA Tree [43], Wanderlust [20], Wishbone [44], SCUBA [45], and SCOUP [46]. According to the benchmarking of different trajectory inference strategies for single-cell data [8], the MST algorithm proved to be a valuable tree construction method that can quickly construct a trajectory depending on the topology of the clusters. CytoTree can perform trajectory construction quickly and simply using MST. Moreover, we present several prevailing methods from clustering to dimensionality reduction. The topology of a trajectory is mainly based on the interrelation of cell clusters, coordinates and dimensions. Therefore, the robustness of the trajectory largely depends on construction methods. CytoTree is flexible in trajectory inference, and provides an optimization step. Trajectory inference through MST according to dimensionality reduction coordinates has been proven to be capable of reproducing known hematopoietic differentiation trajectories (use case 1) and reconstructing a differential trajectory (use case 3). The choice of the tree-shaped trajectory is however still led by biological characteristics and user preferences. Through multiple parameter combinations and adjustments, various options are available for constructing a tree, and users can choose the options that best suit the biological characteristics under study.

## Conclusion

In summary, our comprehensive analysis using both flow and mass cytometry data suggests that CytoTree is flexible in terms of trajectory construction. As the use cases illustrated the utility of CytoTree in facilitating time-course flow cytometry data analysis, CytoTree may be used to better interpret multidimensional single-cell cytometric data in an integrated analysis workflow. We believe that CytoTree can be a valuable tool for applications ranging from clustering and dimensionality reduction to trajectory inference and pseudotime estimation for flow and mass cytometry data analysis.

## Availability and implementation

Project name: CytoTree.

Project home page: https://github.com/JhuangLab/CytoTree, https://bioconductor.org/packages/CytoTree/.

Project tutorial page: https://ytdai.github.io/CytoTree/index.html.

Archived version: 1.0.3.

Operating system(s): Platform independent.

Programming language: R.

Other requirements: None.

License: GPL-3.

## Supplementary Information

**Additional file 1.** Supplementary figures of CytoTree.** Figure S1**–**S8**.

## Data Availability

The datasets generated and/or analysed during the current study are available in the GitHub repository https://github.com/JhuangLab/CytoTree-dataset.

## Abbreviations

SOM: Self-organizing maps
PCA: Principal component analysis
t-SNE: T-distributed stochastic neighbor embedding
UMAP: Uniform manifold approximation and projection
FCS: Flow cytometry standard
MST: Minimum spanning tree
hESC: Human embryonic stem cell
HSC: Hematopoietic stem cell
MPP: Multipotent progenitor
CMP: Common myeloid progenitor
CLP: Common lymphoid progenitor
GMP: Granulocyte–macrophage progenitor
HSPC: Hematopoietic stem/progenitor cell
HPC: Hematopoietic progenitor cell
EHT: Endothelial-to-hematopoietic transition
KNN: K-nearest neighbours
DC: Diffusion component
FMO: Fluorescence minus one
