# Supplementary material for: CytoTree: an R/Bioconductor package for analysis and visualization of flow and mass cytometry data
Source: BMC Bioinformatics. 2021 Mar 22;22:138. doi: 10.1186/s12859-021-04054-2 (PMC7983272; doi:10.1186/s12859-021-04054-2)
Supplement: Supplementary file 1 — Additional file 1. Supplementary figures of CytoTree. Figure S1–S8. [file 12859_2021_4054_MOESM1_ESM.docx]

**Supplementary Material**

**CytoTree: an R/Bioconductor package for analysis and visualization of flow and mass cytometry data**

Yuting Dai^1,†^, Aining Xu^1,†^, Jianfeng Li^1^, Liang Wu^1^, Shanhe Yu^1^, Jun Chen^2^, Weili Zhao^1,*^, Xiao-Jian Sun^1,*^ and Jinyan Huang^1,*^

1. Shanghai Institute of Hematology, State Key Laboratory of Medical Genomics, National Research Center for Translational Medicine at Shanghai, Ruijin Hospital Affiliated to Shanghai Jiao Tong University School of Medicine and School of Life Sciences and Biotechnology, Shanghai Jiao Tong University, 197 Ruijin Er Road, Shanghai 200025, China.
2. Division of Biomedical Statistics and Informatics, Department of Health Sciences Research and Center for Individualized Medicine, Mayo Clinic, 200 1st St SW, Rochester, MN 55905, USA

^﻿†^ Equal contribution

^*^ Corresponding authors: E-mail: huangjy@sjtu.edu.cn (Jinyan H), xjsun@sibs.ac.cn (Xiao-Jian S), zhao.weili@yahoo.com (Weili Z).

**
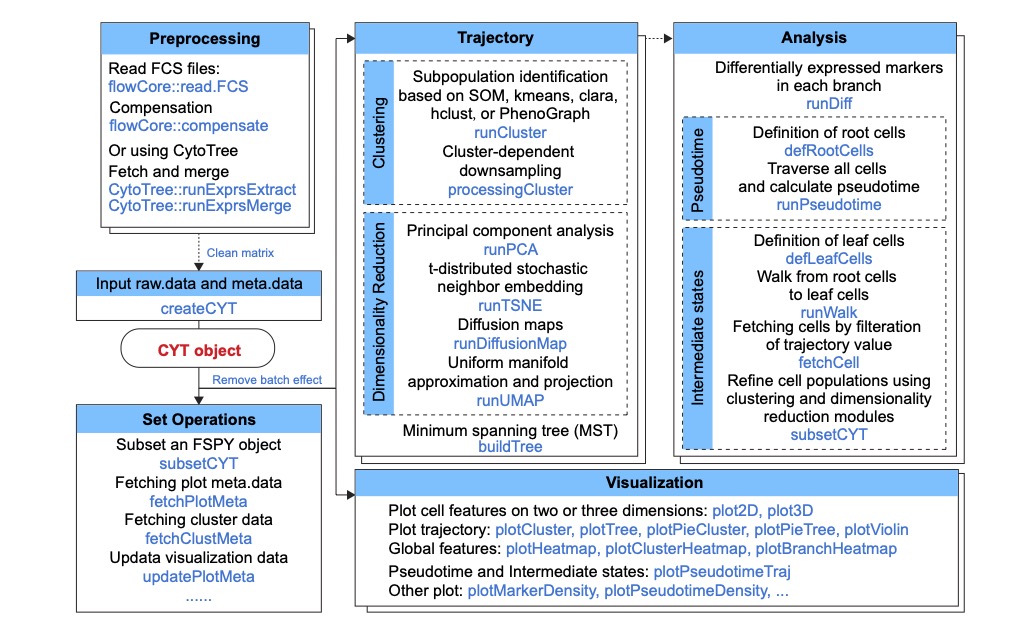
**

**Figure S1. Structure of CytoTree package**

The table headers describe the available modules: preprocessing, trajectory, analysis, visualization and set operations. A short description (black font) and the corresponding function (blue font) are provided for each module. The CytoTree workflow begins with the reading of the FCS data. Compensation, filtration, concatenation and normalization are included in the preprocessing module. A clean matrix after preprocessing is required to build an CYT object, and the analysis workflows of all other modules are all based on the CYT object. The trajectory module contains functions used to perform clustering and dimensionality reduction for cells. The analysis module is based on calculation results from the trajectory module. The visualization module includes functions to generate publication-quality plots from the CYT object. The set operations module includes a function for subsetting an CYT object based on user-defined cells or fetching meta information for clusters and cells during the analysis.

**
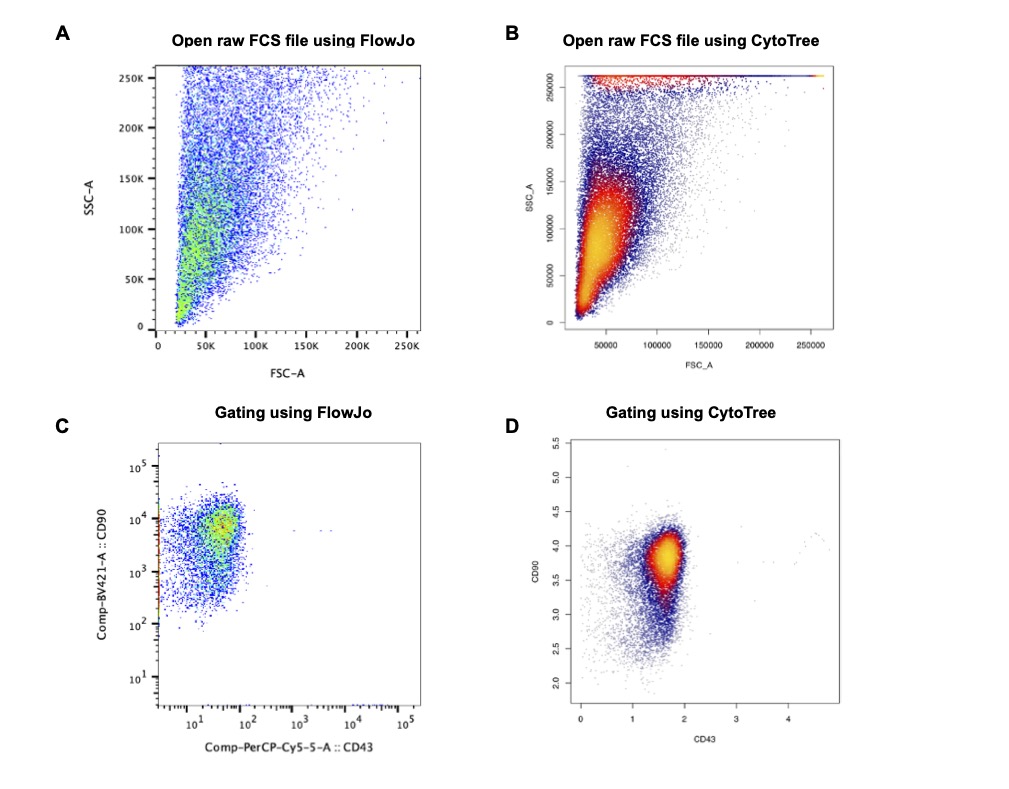
**

**Figure S2. Comparison of scatter plot of FlowJo and CytoTree**

**(A)** Scatter plot when opened raw FCS file using FlowJo. **(B)** Scatter plot when opened raw FCS file using CytoTree. **(C)** Scatter plot using FlowJo after manual gating. **(D)** Scatter plot using CytoTree after manual gating using CytoTree.

**
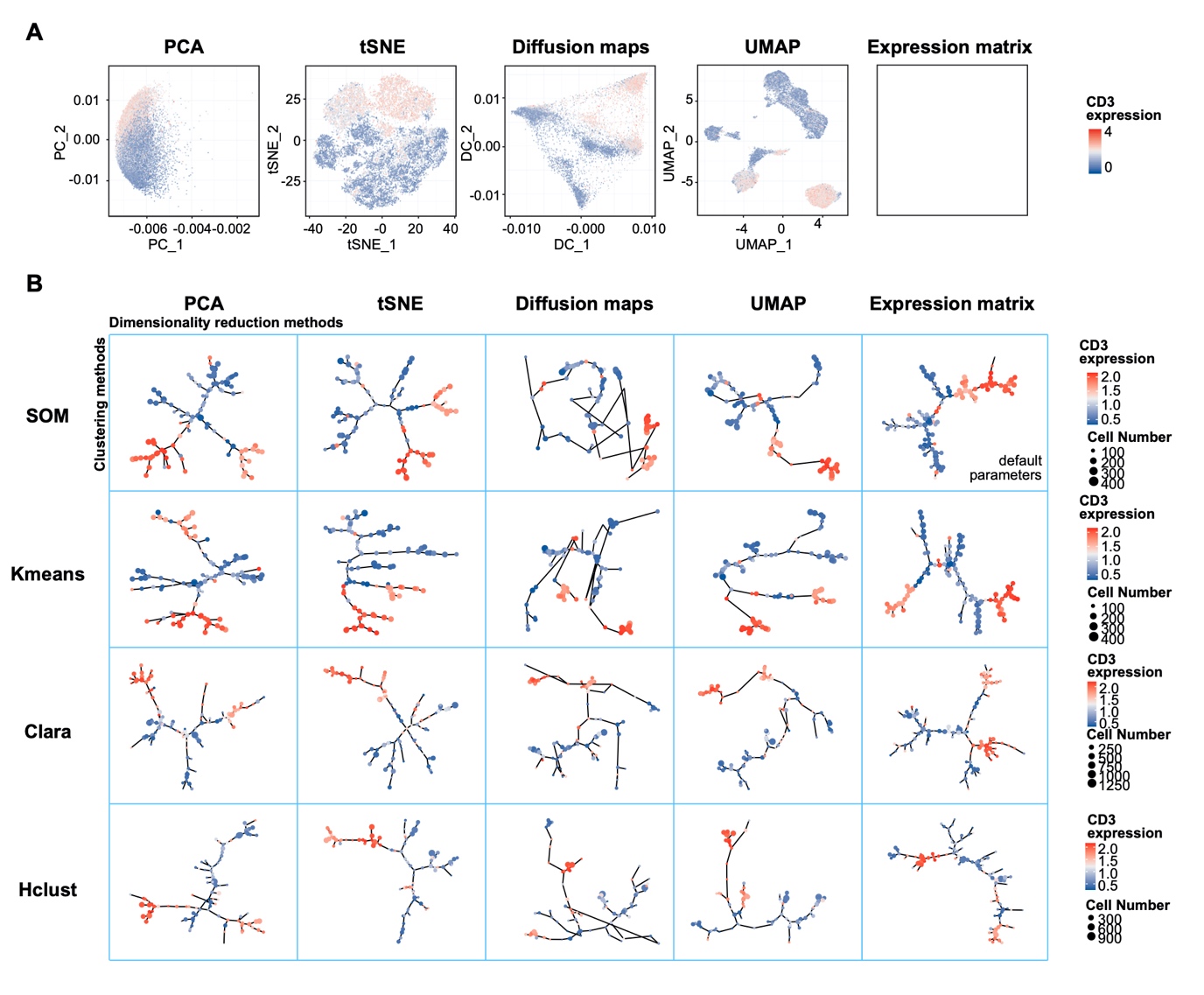
**

**Figure S3. Visualization of trajectory constructed based on different clustering methods and dimensionality reduction methods**

**(A)** The visuality of the four dimensionality reduction methods using the 13-panel mass cytometry data, namely PCA, tSNE, diffusion maps and UMAP. PC_1 and PC_2 represented the first two principal components calculated by PCA, tSNE_1 and tSNE_2 represented the two components calculated by tSNE, DC_1 and DC_2 represented the first two diffusion components calculated by diffusion maps, and UMAP_1 and UMAP_2 represented the two components calculated by UMAP. Color in each point revealed the expression level of CD3. Red color represented a higher expression. **(B)** The trajectory tree constructed using MST based on PCA, tSNE, diffusion maps and UMAP coordinates, and the raw expression matrix, separately. Cluster color was scaled to the mean intensity of the marker expression of the cells within each cluster. Cluster size was scaled based on the cell number of each cluster.

**
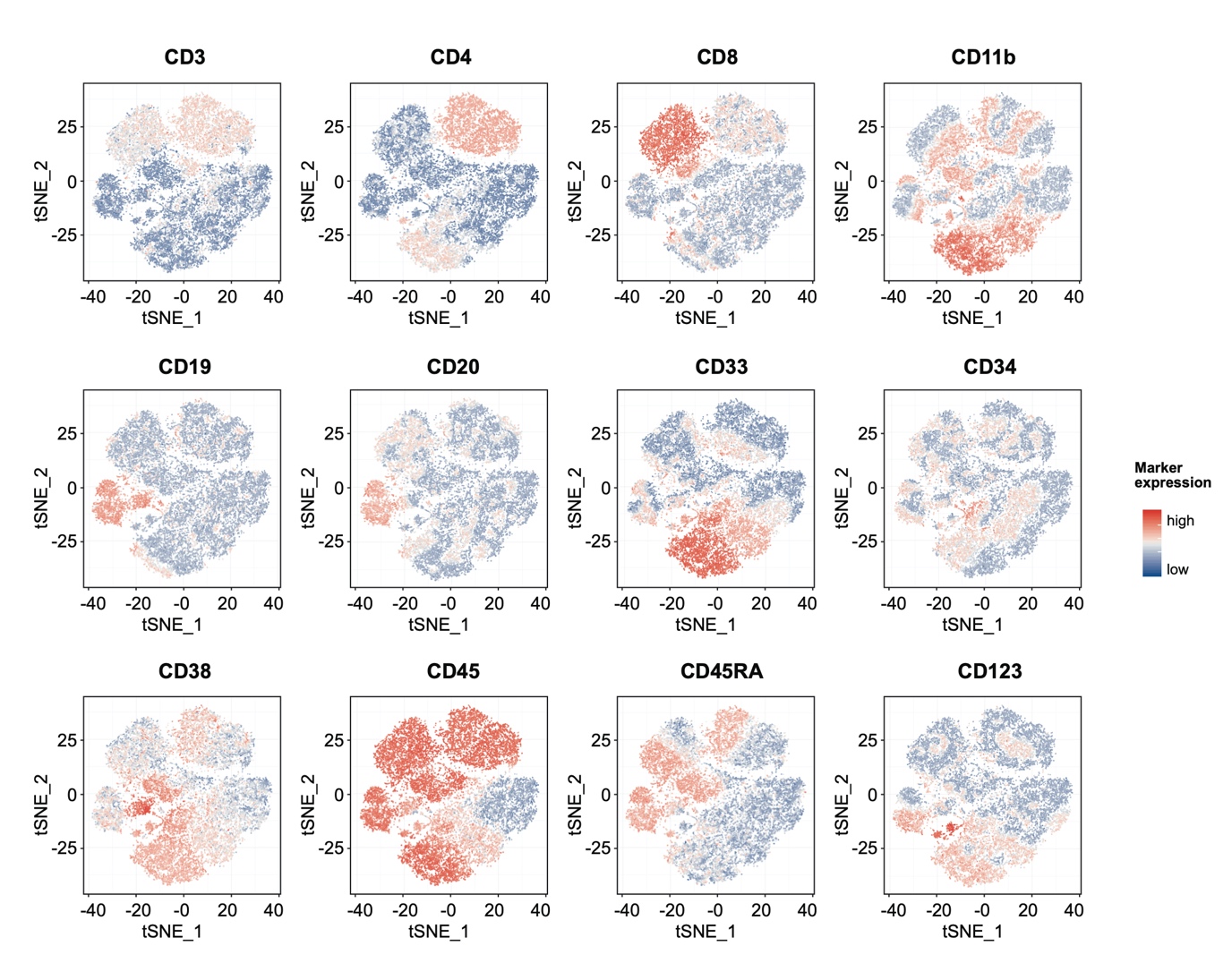
**

**Figure S4. tSNE visualization of marker expression of the 13-panel mass cytometry data**

tSNE plot of the expression level of CD3, CD4, CD8, CD11b, CD19, CD20, CD33, CD34, CD38, CD45, CD45RA and CD123. Color in each point revealed the expression level of each marker. Red color represented a higher expression.

**
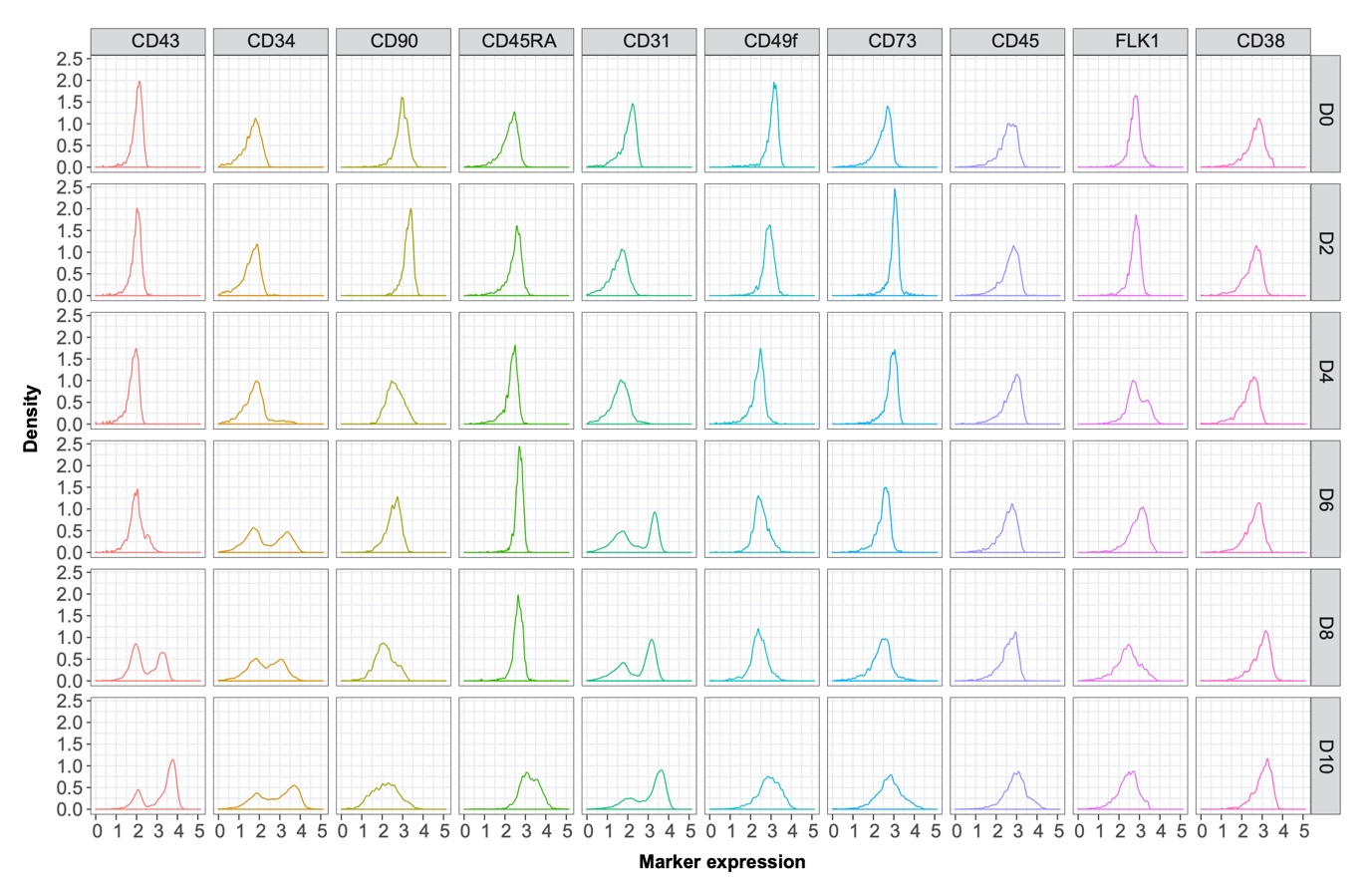
**

**Figure S5. Marker density plot of time-course flow cytometry data**

**
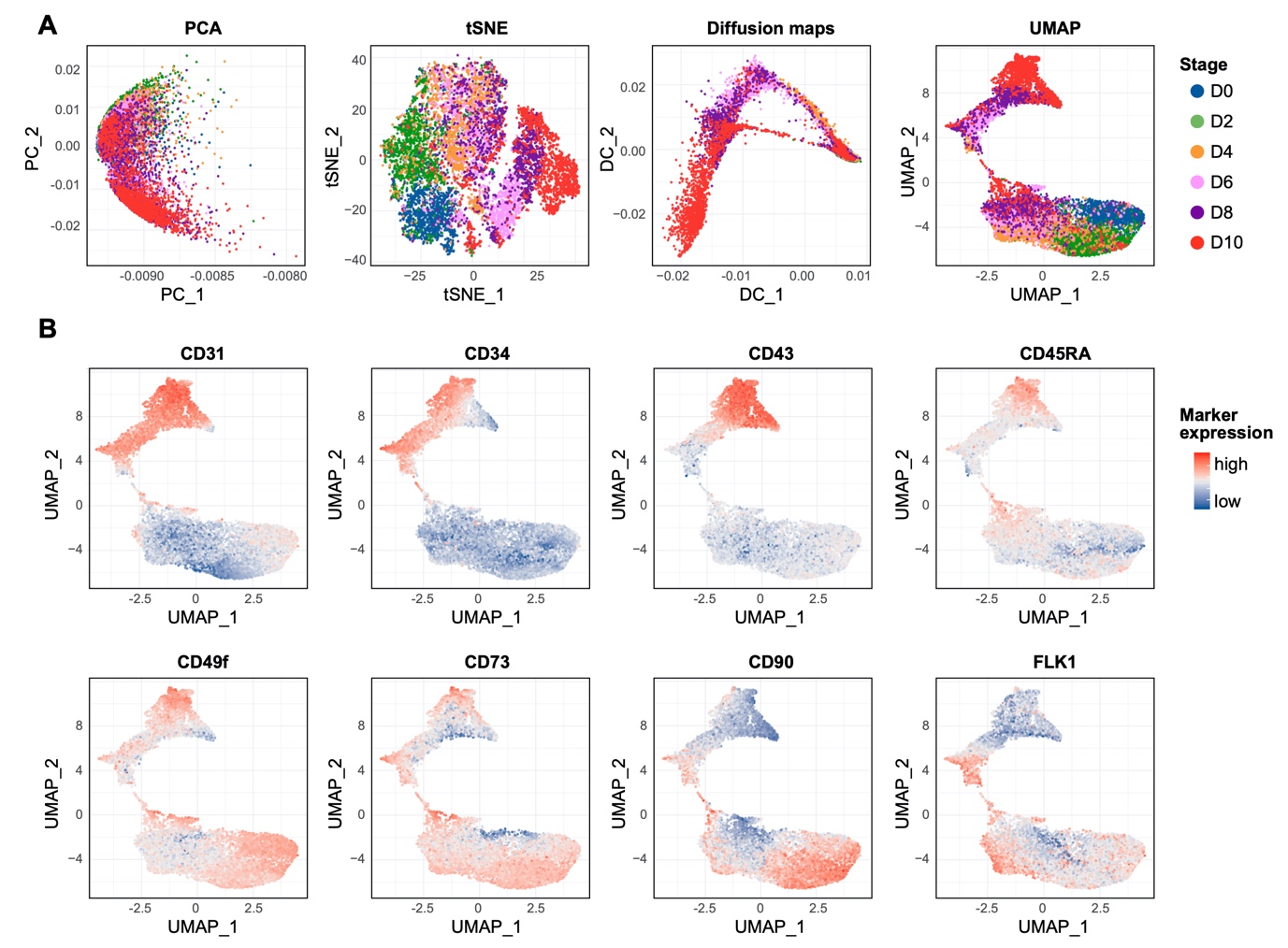
**

**Figure S6. Dimensionality reduction and UMAP visualization of marker expression of the time-course flow cytometry data**

**(A)** The visuality of the first two components calculated by the four dimensionality reduction methods using time-course flow cytometry data: PCA, tSNE, diffusion maps and UMAP. Cells were colored by time point from D0 to D10, separately. **(B)** UMAP plot of the expression level of CD31, CD34, CD43, CD45RA, CD49f, CD73, CD90 and FLK1.

**
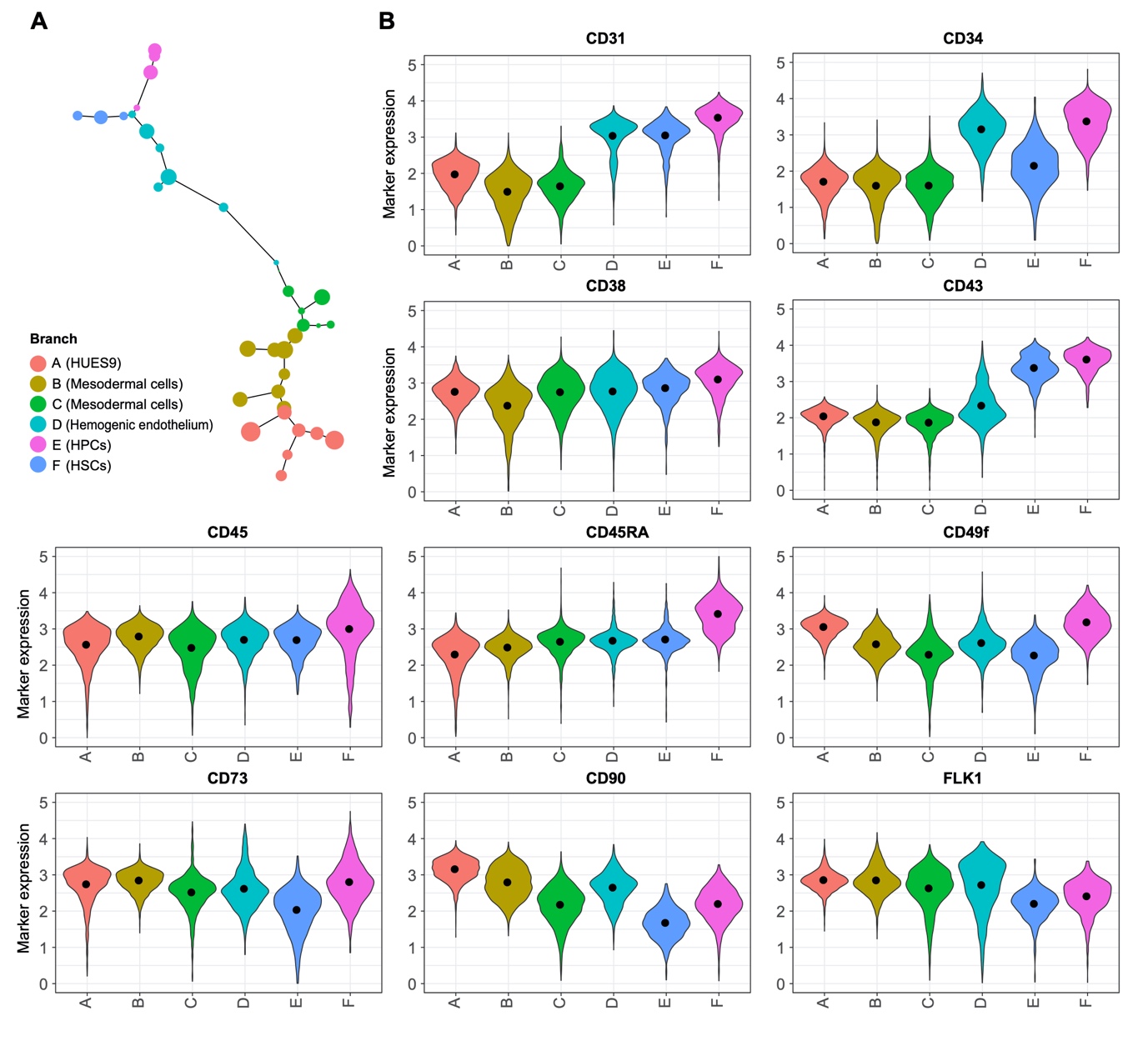
**

**Figure S7. Branch analysis of differentiation trajectory using the time-course flow cytometry data**

**(A)** Branches identified by CytoTree and putative populations were annotated manually according to marker expression. **(B)** Violin plot of the expression level of CD31, CD34, CD38, CD43, CD45, CD45RA, CD49f, CD73, CD90 and FLK1. Each branch was colored different.

**
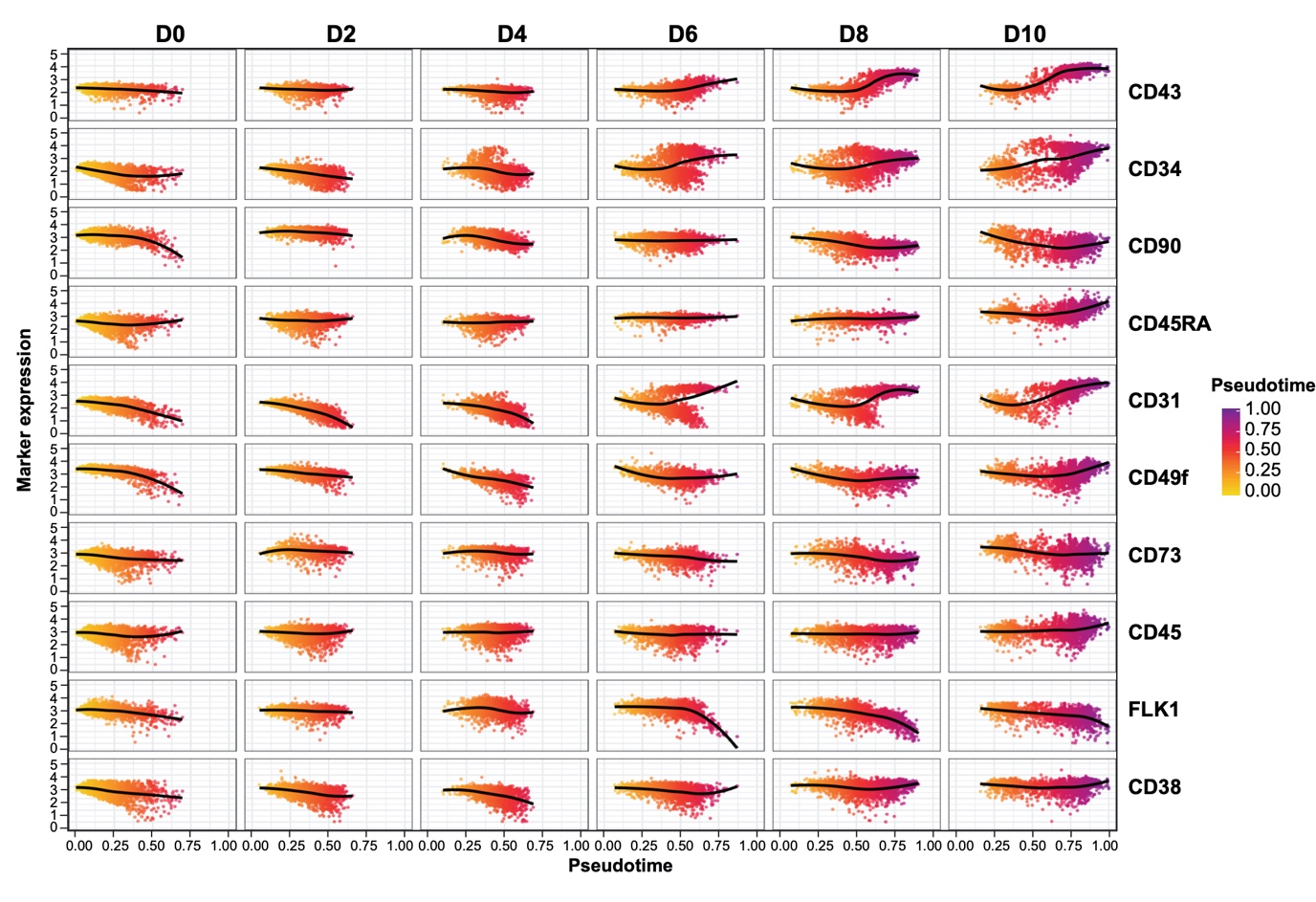
**

**Figure S8. Correlation between markers expression and pseudotime in different time points**
